# Surgical treatment of coexisting intra and extralobar sequestration in an infant

**DOI:** 10.1093/jscr/rjae660

**Published:** 2024-10-17

**Authors:** Ahmad Saleh, Adam Awwad, Ratul Bhattacharyya, Nishith Bhattacharyya

**Affiliations:** School of Medicine, St. George’s University, True Blue, Grenada, West Indies; School of Medicine, St. George’s University, True Blue, Grenada, West Indies; Department of Surgery, St. Joseph's University Medical Center, Patterson, NJ 07503, United States; Division of Pediatric Surgery, St. Joseph's University Medical Center, Patterson, NJ 07503, United States

**Keywords:** intralobar, extralobar, sequestration, pulmonary

## Abstract

Pulmonary sequestration is a rare congenital malformation in which a nonfunctional segment of lung tissue has no communication with the tracheobronchial tree and does not participate in gas exchange. We present a rare case of a 20-month old female with extralobar pulmonary sequestration that was diagnosed at birth. The patient was also found to have a coexisting intralobar sequestration, found during surgery.

## Introduction

Bronchopulmonary sequestration accounts for 0.15 to 6.40% of all congenital lung malformations, thus is an extremely rare congenital malformation [1]. The most common variation of sequestration is intralobar sequestration (ILS). This accounts for 75 to 86% of all cases, and usually presents within the visceral pleura of a healthy lung and drains into the pulmonary vein [2]. Less commonly extralobar sequestration (ELS) is confined to a pulmonary segment characterized by its own visceral pleura and independent venous drainage [3]. While ILS is more often described in adults, ELS is rarely diagnosed in children. The simultaneous occurrence of both is extremely rare in children [1, 4].

## Case report

**Figure 1:**
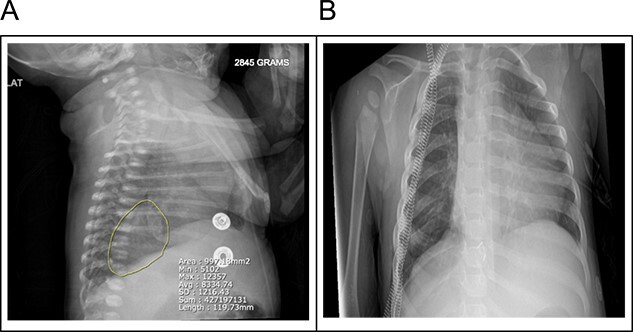
(A) A 3.8 × 2.6 × 2.8 cm ovoid hazy opacity is projecting in the right hemithorax in the expected location of the right lower lobe. (B) Right lower lobe 3.8 cm faintly opaque lesion may represent pulmonary sequestration rather than duplication cyst or other mediastinal lesions.

**Figure 2:**
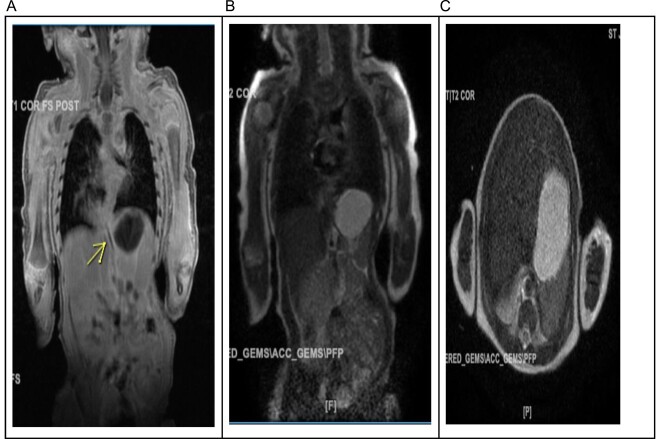
(A) The arterial supply to what appears to be a right lower lobe sequestration arises from the celiac trunk and there may be either a 2nd smaller artery arising more distally or a draining vein to the spleno-portal confluence. (B) The major venous drainage appears to extend to the right inferior pulmonary vein. (C) In the right lower lobe there is a 2.9 × 2.6 cm mass containing internal vascular architecture and suggestive of a sequestration.

A 20-month female presented with her mother to the pediatric surgery office with a known history of pulmonary sequestration. Imaging at the time of birth revealed a 3.8 × 2.6 × 2.8 cm ovoid hazy opacity projecting in the right hemithorax in the expected location of the right lower lobe (Fig. 1). This was followed up with an MRI confirming a 2.9 × 2.6 cm segment of ELS arterial inflow identified from the celiac trunk (Fig. 2). No emergent surgeries were indicated at that time.

Shortly after the office visit, the child was readmitted for a short course treatment of a right upper lobe pneumonia. At this time it was decided to revisit the finding of sequestration, and proceed with surgical treatment.

**Figure 3:**
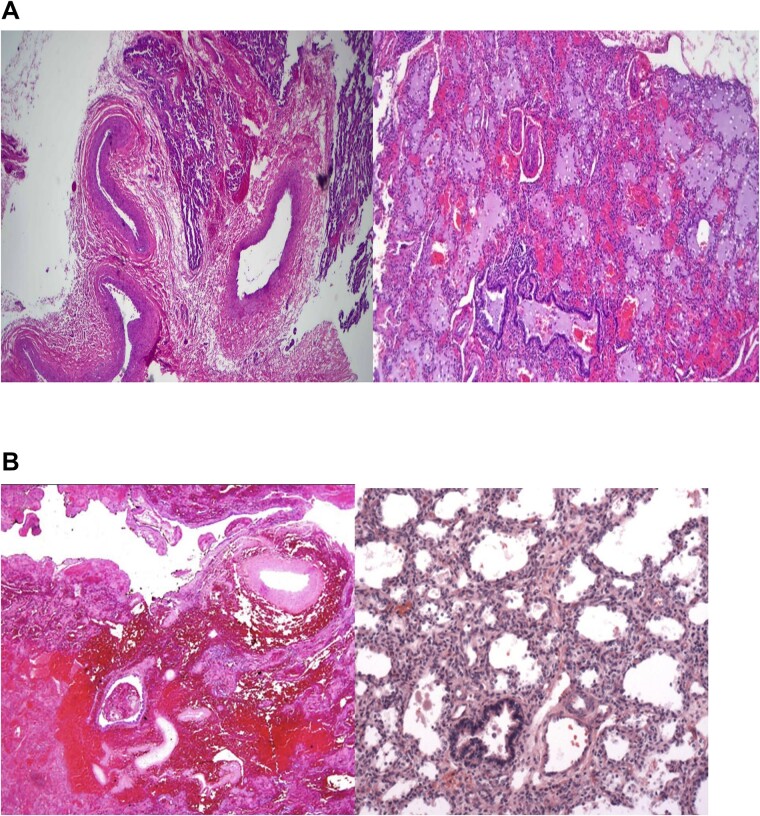
(A) Sections show lung tissue with thick walled large vessels in the septa and near the hilum representing a systemic arterial supply. These findings are consistent with ILS. (B) Hemorrhagic lung parenchyma that was covered by pleura, supplied by thick walled large vessels and clinically located outside the lung thus satisfying the criteria for ELS.

A right lower lobectomy for removal of ELS was performed. Two separate biopsies were obtained, which revealed sections of lung tissue with thick-walled, large vessels in the septa and near the hilum (Fig. 3). This was indicative of a systemic arterial supply, consistent with ILS. The lung tissue also exhibited mild to moderate chronic bronchitis and areas of alveolar collapse, with no cyst formation or mucinous metaplasia (Fig. 3). Hilar lymph nodes were unremarkable. The other biopsy displayed lung tissue with thick-walled blood vessels and interstitial septa, consistent with ELS (Fig. 3). Postoperative recovery and follow up have been unremarkable.

## Discussion

Although the etiology of pulmonary sequestration is not fully understood, the most accepted theory involves an accessory embryological lung bud that forms caudally to the normal pulmonary bud. The accessory derives its blood supply through splanchnic vessels forming its own visceral pleura and resulting as an extralobar segment [5]. Early surgical interventions are recommended around age 10–12 months to prevent complications [6]. The most common complications in infancy and childhood are growth abnormalities and recurrent infections. Pseudomonas is the most common microbe affiliated with these episodes [7].

Surgical resection is the preferred method of treatment [8]. This is the only option involving curative elimination of diseased tissues. Thus avoiding the patient such complicated sequelae as persistent infection and cystic malformation. The major complexities associated with such invasive avenues as surgical resection include scarring and adhesion formation. These sequelae can cause discomfort yet are mainly restricted to self-limiting circumstances. In select cases, a conservative management strategy may be adopted, eschewing invasive interventions. However such approaches are unable to provide spontaneous or prolonged resolution.

## Conclusion

In conclusion, this case exemplifies the importance of vigilant monitoring and timely intervention for pediatric patients with pulmonary sequestration. The diagnostic journey from initial imaging to surgical management highlights the complexities involved in treating this condition. The discrepancy between preoperative imaging and histological findings highlights the necessity for further evaluation, such as tissue biopsy. Pulmonary sequestration is an important differential diagnosis in pediatric patients presenting with recurrent respiratory issues. Early surgical intervention can be preventative in further clinical complications such as growth retardation and recurrent infection. Elective intervention is imperative in ensuring favorable outcomes and uneventful postoperative recovery. This case adds valuable insight toward the general management of our understanding of pulmonary sequestration. Our case shows the importance of considering surgical intervention in a timely manner.
